# Supplementary material for: Splice-Junction-Based Mapping of Alternative Isoforms in the Human Proteome
Source: Cell Rep. Author manuscript; Available in PMC 2020 Jan 15. (PMC6961840; doi:10.1016/j.celrep.2019.11.026)
Supplement: 2 [file NIHMS1546469-supplement-2.zip › DF1/PXD009737/TestisTrypsin/TestisTrypsin_69_TBC1D21_LAITLHVTFR.pdf]

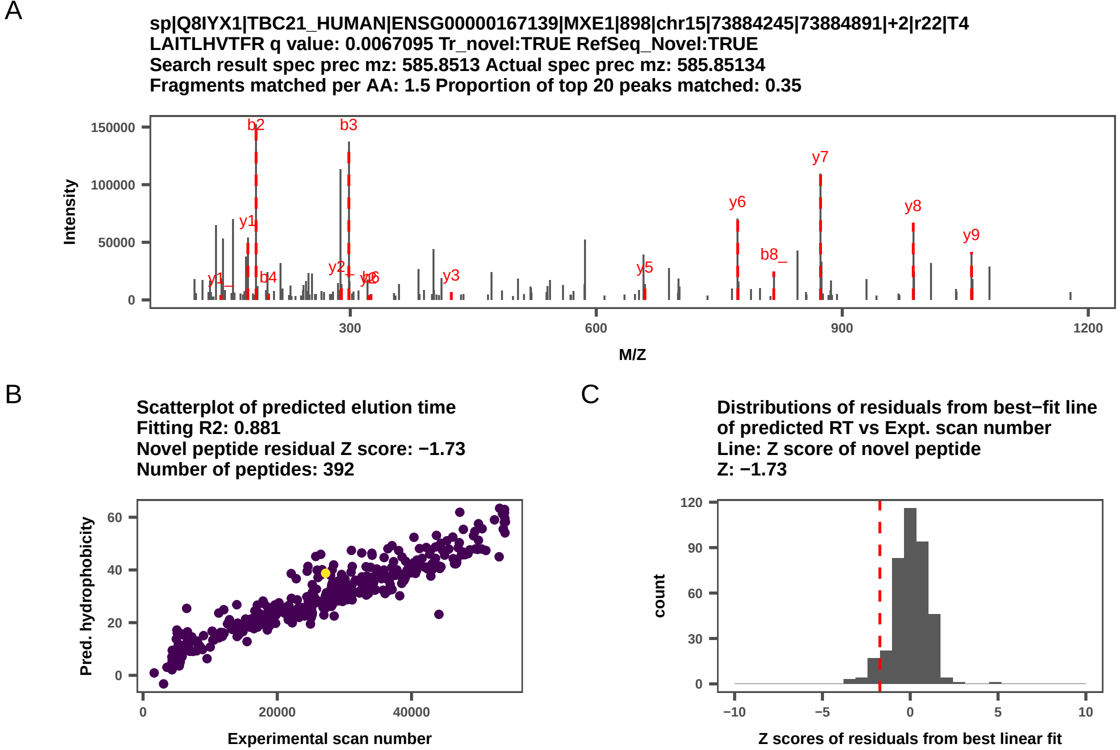
A
sp|Q8IYX1|TBC21_HUMAN|ENSG00000167139|MXE1|898|chr15|73884245|73884891|+2|r22|T4
LAITLHVTFR q value: 0.0067095 Tr_novel:TRUE RefSeq_Novel:TRUE
Search result spec prec mz: 585.8513 Actual spec prec mz: 585.85134
Fragments matched per AA: 1.5 Proportion of top 20 peaks matched: 0.35
Intensity
150000
100000
50000
0
b2
b3
y7
y6
y8
y9
b8_
y5
y1
y1_
b4
y2
y3
M/Z
300
600
900
1200
B
Scatterplot of predicted elution time
Fitting R2: 0.881
Novel peptide residual Z score: −1.73
Number of peptides: 392
Pred. hydrophobicity
60
40
20
0
Experimental scan number
0
20000
40000
C
Distributions of residuals from best−fit line
of predicted RT vs Expt. scan number
Line: Z score of novel peptide
Z: −1.73
count
120
90
60
30
0
Z scores of residuals from best linear fit
−10
−5
0
5
10
